# Polymyositis-Like Myopathy With Anti-PL-12 Antibody Positivity and Coexisting Antiphospholipid Syndrome: Diagnostic and Management Challenges in the Absence of Systemic Features

**DOI:** 10.7759/cureus.78002

**Published:** 2025-01-26

**Authors:** Andres D Parga, Irfan Raheem

**Affiliations:** 1 Medicine/Rheumatology, Hospital Corporation of America (HCA) Westside Regional Medical Center, Plantation, USA

**Keywords:** antiphospholipid syndrome, anti-pl-12 antibody, anti-synthetase syndrome, autoimmune myopathies, clinical dermatology, clinical rheumatology, immunosuppressive therapy, polymyositis, polymyositis-like inflammatory myopathy, polymyositis-like myopathy

## Abstract

Polymyositis-like inflammatory myopathies are a rare subset of idiopathic inflammatory myopathies (IIMs) characterized by proximal muscle weakness, elevated muscle enzymes, and immune-mediated skeletal muscle damage. These conditions are often associated with myositis-specific autoantibodies (MSAs), such as anti-alanyl-tRNA synthetase antibody (anti-PL-12), which define subtypes like anti-synthetase syndrome. We present the case of a 50-year-old female patient with progressive proximal muscle weakness, elevated creatine kinase and aldolase levels, and serological evidence of anti-PL-12 antibodies. Muscle biopsy revealed hallmark findings of immune-mediated myopathy, including increased sarcolemmal major histocompatibility complex class I (MHC1) expression, vascular membrane attack complex (C5b-9) deposition, and focal myofasciitis. Despite the absence of hallmark systemic features of the anti-synthetase syndrome, such as interstitial lung disease (ILD) or arthritis, these findings confirmed a diagnosis of polymyositis-like myopathy within the anti-synthetase syndrome spectrum.

The patient’s concurrent antiphospholipid syndrome (APS) necessitated careful anticoagulation management while initiating immunosuppressive therapy with prednisone and mycophenolate mofetil, which led to clinical and biochemical improvement. This case underscores the diagnostic challenges posed by incomplete phenotypes of anti-synthetase syndrome and the critical role of muscle biopsy in confirming autoimmune myopathies. By illustrating the variability of presentations and the necessity of a multidisciplinary approach, this report contributes to the understanding and management of complex autoimmune myopathies.

## Introduction

Idiopathic inflammatory myopathies (IIMs) represent a rare and heterogeneous group of autoimmune disorders characterized by immune-mediated muscle inflammation and damage. Idiopathic inflammatory myopathies, including polymyositis and anti-synthetase syndrome, have an estimated prevalence of 10-20 cases per 100,000 people. Polymyositis itself is less common, particularly in its pure form without overlapping features. Anti-synthetase syndrome, associated with autoantibodies like anti-alanyl-tRNA synthetase antibody (anti-PL-12), accounts for 20% to 30% of IIM cases, with a higher prevalence in females and a typical onset in middle age. Polymyositis is defined by proximal muscle weakness, elevated serum muscle enzymes, and characteristic histopathological findings. Polymyositis is further distinguished from other myopathies by the absence of cutaneous manifestations seen in dermatomyositis or degenerative features characteristic of inclusion body myositis (IBM) [1]. Despite these distinctions, overlaps and atypical presentations complicate diagnosis and management.

Myositis-specific autoantibodies (MSAs) have revolutionized the understanding of IIMs by providing insights into disease subsets, pathophysiology, and associated systemic features. Anti-synthetase syndrome, a subtype of IIM, is defined by the presence of anti-synthetase antibodies such as anti-PL-12, which target alanyl-tRNA synthetase. This syndrome is typically characterized by a constellation of clinical features, including interstitial lung disease (ILD), arthritis, Raynaud’s phenomenon, mechanic’s hands, and myositis [2]. However, atypical presentations lacking hallmark systemic manifestations pose diagnostic challenges, necessitating a high degree of clinical suspicion.

This case report focuses on a 50-year-old female patient presenting with a polymyositis-like myopathy in the context of anti-PL-12 antibody positivity. Notably, her presentation was devoid of hallmark systemic features of anti-synthetase syndrome, such as ILD or arthritis. This case is further complicated by the coexistence of antiphospholipid syndrome (APS), a prothrombotic condition requiring long-term anticoagulation. The presence of multiple autoantibodies, including cytosolic 5' nucleotidase 1A and anti-Sjögren's syndrome-related antigen A (anti-Ro52), highlights the complexity of autoimmune overlap syndromes and raises important questions regarding disease pathogenesis and clinical variability.

Muscle biopsy remains the diagnostic gold standard in IIMs, particularly in cases with atypical clinical or serological findings [3]. In this patient, histopathological analysis revealed hallmark features of immune-mediated myopathy, including increased sarcolemmal expression of major histocompatibility complex class I (MHC1), vascular deposition of membrane attack complex (C5b-9), and focal myofasciitis. These findings were critical in confirming the diagnosis and guiding targeted immunosuppressive therapy.

This report aims to expand the understanding of polymyositis-like inflammatory myopathies within the spectrum of anti-synthetase syndrome. By presenting this case, we emphasize the importance of a multidisciplinary diagnostic approach, the role of muscle biopsy in confirming autoimmune myopathies, and the therapeutic challenges posed by comorbid conditions such as APS. Additionally, we discuss the clinical implications of overlapping autoantibodies and their role in disease heterogeneity, with the goal of advancing diagnostic and therapeutic strategies for IIMs.

## Case presentation

A 50-year-old female patient presented with a one-year history of progressive proximal muscle weakness, primarily affecting her shoulders and hips. She reported difficulty performing activities of daily living, such as lifting objects, combing her hair, and climbing stairs. The patient denied associated muscle pain, fatigue, dysphagia, or respiratory symptoms. Notably, there were no systemic features such as fever, weight loss, rash, or Raynaud’s phenomenon. She also described mild joint discomfort in the shoulders and hands without swelling, erythema, or prolonged morning stiffness. Her gradual onset and progressive symptoms prompted further evaluation. Classic signs of dermatomyositis, including Gottron's papules, heliotrope rash, and the shawl sign, were absent in this patient, further supporting a diagnosis of polymyositis-like myopathy rather than classic dermatomyositis. This absence, despite some overlapping histopathological features, emphasized the importance of integrating clinical findings with biopsy results.

The patient’s past medical history included hypertension, hypothyroidism, obesity (body mass index (BMI): 47.9 kg/m^2^), and APS, diagnosed two years prior following an episode of deep vein thrombosis (DVT). She was on long-term anticoagulation therapy with apixaban at the time of the DVT. Her surgical history included a partial hysterectomy and gastric sleeve surgery. She denied a family history of autoimmune or rheumatologic diseases, as well as smoking, alcohol consumption, or illicit drug use.

On physical examination, the patient appeared well-nourished but obese, with stable vital signs. Muscle strength testing revealed symmetric proximal weakness, graded 4/5 in the shoulder and hip girdles bilaterally. There was no muscle atrophy, fasciculations, or contractures. A detailed joint examination revealed no evidence of synovitis, swelling, or tenderness. Skin examination was unremarkable, with no rash, mechanic’s hands, or nail fold abnormalities. Cardiopulmonary and neurological evaluations were normal. Examination findings are summarized in (Table 1).

**Table 1:** The patient's physical examination findings

System	Findings
General	Well-nourished, obese, no acute distress
Neurological	Symmetric proximal muscle weakness (4/5 strength) in shoulder and hip girdles
Musculoskeletal	No muscle atrophy, contractures, or synovitis
Skin	No rashes, ulcers, mechanic’s hands, or nail fold abnormalities
Cardiopulmonary	Clear breath sounds; normal heart sounds
Abdomen	Normal; no organomegaly or tenderness

Initial laboratory studies revealed elevated muscle enzymes and markers of systemic inflammation. Additionally, serological testing identified multiple myositis-specific and associated autoantibodies. Key laboratory findings are detailed in (Table 2). The findings, including elevated creatine kinase, anti-PL-12 antibody positivity, and muscle biopsy results demonstrating increased sarcolemmal MHC1 expression and vascular deposition of C5b-9, aligning closely with the pathophysiology of polymyositis-like myopathies and anti-synthetase syndrome. These conditions are characterized by immune-mediated muscle inflammation triggered by dysregulated adaptive immunity. Increased sarcolemmal MHC1 expression is a hallmark of immune-mediated myopathies, indicating the upregulation of antigen-presenting pathways in muscle fibers. This aberrant expression facilitates the activation of cytotoxic CD8+ T-cells, which directly target myofibers, leading to muscle damage. The vascular deposition of C5b-9, the terminal component of the complement cascade, suggests complement-mediated endothelial injury, further contributing to the inflammatory process. Anti-PL-12 antibodies, specific to alanyl-tRNA synthetase, play a central role in anti-synthetase syndrome by disrupting cellular homeostasis and triggering immune responses. These antibodies target cytoplasmic components released during cellular stress or damage, resulting in the formation of immune complexes and the recruitment of inflammatory cells. This process exacerbates tissue injury and contributes to the systemic manifestations often observed in anti-synthetase syndrome, although this case notably lacked hallmark features such as ILD or arthritis [4].

**Table 2:** The patient's laboratory findings

Test	Result	Reference range	Interpretation
Creatine hinase (CK)	341 U/L	<92 U/L	Elevated, indicating muscle damage
Aldolase	15 U/L	<8.1 U/L	Elevated, suggesting active muscle inflammation
Erythrocyte sedimentation rate (ESR)	58 mm/h	<20 mm/h	Elevated, reflecting systemic inflammation
C-reactive protein (CRP)	8.7 mg/L	<8.0 mg/L	Elevated, indicative of inflammation
Antinuclear antibody-HEp-2 (ANA by HEp-2)	1:320	Negative	Positive, speckled nuclear and cytoplasmic patterns
Anti-alanyl-tRNA synthetase antibody (Anti-PL-12)	Positive	Negative	Specific for anti-synthetase syndrome
Anti-scleroderma 70 immunoglobulin G (Anti-Scl-70 IgG)	8.6	Negative	Equivocal
Anti-Sjögren's syndrome-related antigen A immunoglobulin G (Anti-Ro52 IgG)	11.0	Negative	Positive
Antibodies to U1 ribonucleoprotein particle immunoglobulin G (Anti-U1RNP IgG)	7.4	Negative	Equivocal
Anti-cardiolipin immunoglobulin A (ACA)	22.0	Negative	Positive
Beta-2-microglobulin (B2M)	3.31	<2.5 mg/L	Elevated, consistent with immune activation
Kappa light chain	72.4	3.3–19.4 mg/L	Elevated
Lambda light chain	43.3	5.7–26.3 mg/L	Elevated
Immunoglobulin A (IgA)	517 mg/dL	47–310 mg/dL	Elevated, reflecting immune activation
Immunoglobulin G (IgG)	2437 mg/dL	600–1640 mg/dL	Elevated, consistent with immune dysregulation

**Figure 1:**
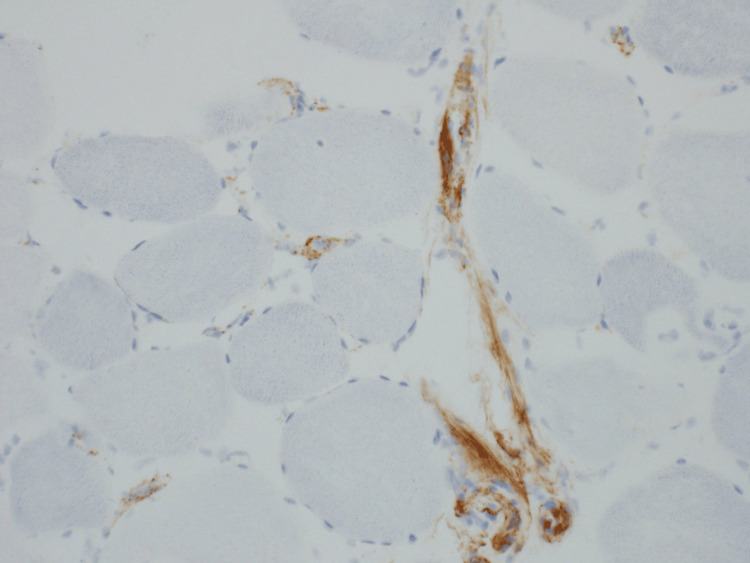
C5b-9 vascular deposition Histopathology of skeletal muscle biopsy showing membrane attack complex (C5b-9) deposition in the vasculature (muddy brown-orange). Immunohistochemical staining demonstrates prominent vascular deposition, consistent with complement activation and immune-mediated injury. Original magnification: 200x; Image courtesy: Dr. T. David Bourne and Arkana Laboratories

**Figure 2:**
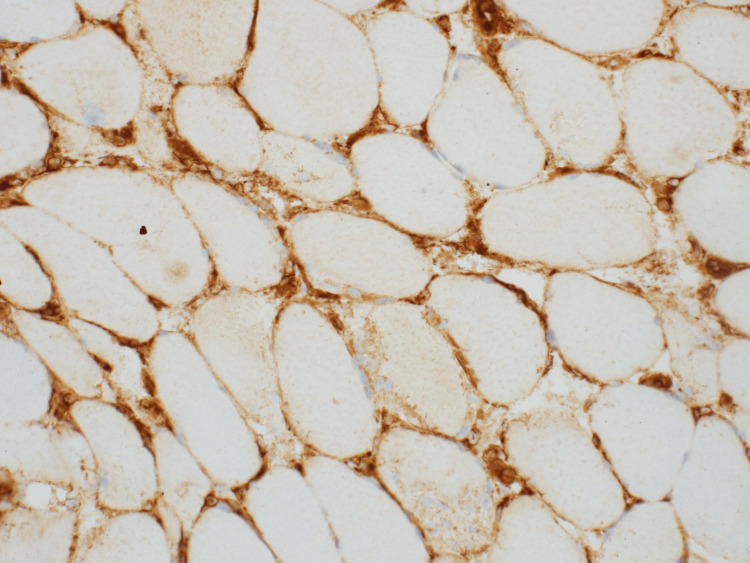
MHC1 with carcolemmal staining Histopathology of skeletal muscle biopsy showing major histocompatibility complex class I (MHC1) upregulation with sarcolemmal staining (brownish, granular, linear staining pattern along the sarcolemmal membrane of the muscle fibers). Immunohistochemical staining demonstrates diffuse and uniform expression of MHC1 along the sarcolemmal membrane of muscle fibers, consistent with immune-mediated muscle injury. Original magnification: 200x; Image courtesy: Dr. T. David Bourne and Arkana Laboratories

A muscle biopsy of the right lower leg was performed to confirm the diagnosis. Histopathological analysis revealed hallmark features of immune-mediated myopathy (Figures 1-2) and further summarized in (Table 3).

**Table 3:** The patient's muscle biopsy findings MHC1: major histocompatibility complex class I; C5b-9: membrane attack complex

Biopsy component	Key findings
Fiber size variation	Moderate, with necrotic and regenerating fibers
Endomysial inflammation	Patchy mononuclear infiltrates, predominantly T-cells
Sarcolemmal MHC1 expression	Increased, consistent with immune-mediated injury (Figure 2)
Vascular involvement	C5b-9 deposition in capillaries (Figure 1)
Epimysial connective tissue	Fibrinopurulent debris, suggestive of focal myofasciitis

The biopsy findings confirmed a diagnosis of polymyositis-like inflammatory myopathy associated with anti-PL-12 antibody positivity. Importantly, no features of inclusion body myositis, such as rimmed vacuoles or abnormal cytoplasmic inclusions, were observed.

The patient was initiated on prednisone 10 mg daily and mycophenolate mofetil 500 mg twice daily to suppress immune-mediated inflammation and minimize corticosteroid-related side effects. A gradual approach with prednisone 10 mg daily was chosen to minimize the risk of corticosteroid-related side effects, particularly in the context of the patient’s comorbidities, including obesity and APS, which increases the risk of thrombotic complications. Additionally, the concurrent use of mycophenolate mofetil as a steroid-sparing agent was intended to provide effective long-term immunosuppression while reducing the need for high-dose corticosteroids. Due to her APS, she continued anticoagulation. Physiotherapy was initiated to improve strength and functionality. At her one-month follow-up, she reported significant improvement in proximal muscle strength, and her laboratory studies demonstrated normalization of creatine kinase and aldolase levels, as well as decreased inflammatory markers. The biopsy site showed complete healing without delayed closure or infection.

This case underscores the diagnostic complexity and therapeutic challenges associated with autoimmune myopathies, particularly when presenting with atypical or incomplete phenotypes.

## Discussion

Polymyositis-like inflammatory myopathies, a rare subset of IIMs, present significant diagnostic and therapeutic challenges. These autoimmune disorders are characterized by proximal muscle weakness, elevated muscle enzymes, and immune-mediated muscle damage, with variability in their clinical presentations [5]. This case illustrates the intricacies of diagnosing and managing a polymyositis-like myopathy associated with anti-PL-12 antibodies, highlighting its overlap with anti-synthetase syndrome and the complexity introduced by comorbid conditions.

Diagnostic importance of histopathology and autoantibody profiling

Muscle biopsy remains the cornerstone of diagnosing IIMs, particularly in atypical presentations. In this patient, histopathological findings, including moderate fiber size variation, necrotic and regenerating fibers, patchy mononuclear infiltrates, and increased sarcolemmal MHC1 expression, provided definitive evidence of immune-mediated myopathy [6]. The presence of vascular deposition of C5b-9 further supported an autoimmune etiology, ruling out other potential diagnoses such as inclusion body myositis, which typically features rimmed vacuoles and cytoplasmic inclusions [7].

The biopsy also revealed fibrinopurulent debris in the epimysium, suggestive of focal myofasciitis [8]. While not commonly associated with anti-synthetase syndrome, this finding underscores the spectrum of inflammatory changes possible in polymyositis-like conditions. These histological features were essential in integrating serological and clinical findings to reach a conclusive diagnosis.

Serological analysis revealed a multifaceted autoantibody profile, including anti-PL-12 antibodies specific to anti-synthetase syndrome, cytosolic 5' nucleotidase 1A antibodies often linked to IBM, and anti-Ro52 antibodies associated with systemic autoimmune conditions. The presence of anti-PL-12 antibodies, despite the absence of hallmark systemic features such as ILD, underscores the phenotypic heterogeneity within anti-synthetase syndrome. The coexistence of cytosolic 5' nucleotidase 1A antibodies further complicated the diagnostic process, as they are typically associated with degenerative myopathies rather than inflammatory ones [9].

Elevated levels of beta-2-microglobulin, kappa, and lambda light chains, as well as increased immunoglobulin A (IgA) and immunoglobulin G (IgG), pointed toward systemic immune activation and dysregulation. Beta-2-microglobulin, a marker of immune activation, has been implicated in various autoimmune diseases and could reflect chronic inflammation in this patient [10]. Similarly, the elevated kappa and lambda light chains and immunoglobulins highlight potential humoral immune contributions to the pathogenesis of this condition. Additionally, the elevated beta-2 microglobulin, kappa and lambda light chains, and immunoglobulin levels raise potential concerns for an underlying monoclonal gammopathy or broader immune dysregulation. While these markers can reflect systemic inflammation associated with autoimmune myopathies, their presence warrants further investigation to rule out coexisting conditions such as a monoclonal gammopathy of undetermined significance (MGUS) or a plasma cell dyscrasia. Additional tests, including serum protein electrophoresis (SPEP) and immunofixation, may be considered to evaluate these possibilities. These findings suggest broader systemic immune involvement beyond skeletal muscle inflammation, further complicating the disease phenotype.

The equivocal results for anti-scleroderma 70 (anti-Scl-70) and antibodies to U1 ribonucleoprotein particle (anti-U1RNP) highlight potential overlaps with other autoimmune conditions, such as systemic sclerosis or mixed connective tissue disease [11]. However, their relevance is limited without additional clinical or serological evidence supporting these diagnoses, making their role in this case less definitive.

**Figure 3:**
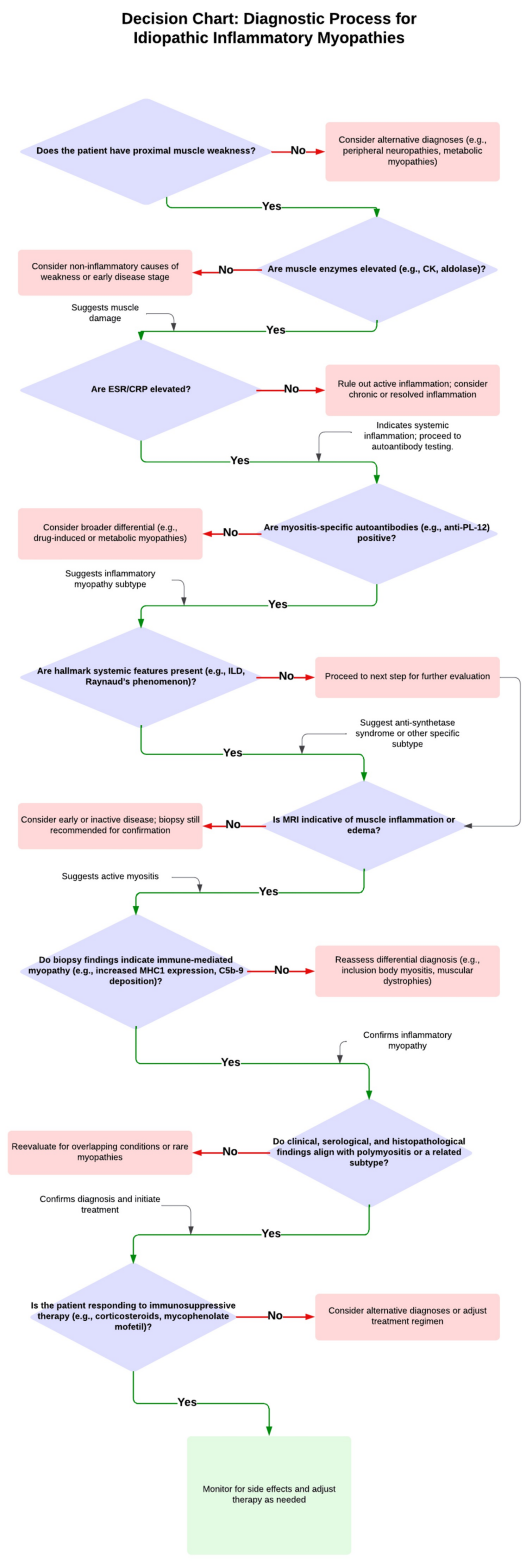
Decision chart for the diagnostic process of idiopathic inflammatory myopathies This flowchart outlines a systematic approach to diagnosing idiopathic inflammatory myopathies, particularly polymyositis-like conditions. The decision-making process incorporates clinical presentation, laboratory evaluations, imaging studies, and muscle biopsy findings to confirm the diagnosis and guide management. Key diagnostic steps include assessing proximal muscle weakness, elevated muscle enzymes, inflammatory markers, and myositis-specific autoantibodies, followed by histopathological confirmation. The chart also highlights the integration of clinical, serological, and histopathological data to achieve a comprehensive diagnosis. An MRI is critical for detecting muscle inflammation and guiding biopsies, especially in incomplete phenotypes. EMG helps confirm muscle involvement and exclude neuropathic causes. An MRI is prioritized for biopsy guidance, while EMG is used if MRI findings are inconclusive. In atypical cases, imaging helps refine the diagnostic approach and expedite biopsy. ESR: erythrocyte sedimentation rate; CRP: C-reactive protein; ILD: interstitial lung disease; EMG: electromyography; anti-PL-12: anti-alanyl-tRNA synthetase antibody; MHC1: major histocompatibility complex class I; C5b-9: membrane attack complex Adapted from [12, 13]

To illustrate the diagnostic approach for idiopathic inflammatory myopathies, particularly polymyositis-like conditions, a decision chart summarizing key steps is presented below (Figure 3).

Implications of an incomplete phenotype

The absence of hallmark systemic features of anti-synthetase syndrome, such as ILD, mechanic’s hands, and arthritis, complicates the diagnostic process. Interstitial lung disease is a predominant cause of morbidity and mortality in anti-synthetase syndrome, and its absence in this case may suggest a more favorable prognosis [14]. However, the strong association of anti-PL-12 antibodies with ILD necessitates ongoing surveillance for potential pulmonary involvement, particularly given the possibility of subclinical or delayed-onset disease. Regular pulmonary function tests (PFTs) every six to 12 months and high-resolution computed tomography (HRCT) as needed are recommended to monitor for emerging ILD [15].

The lack of systemic manifestations, while simplifying initial management, underscores the need for vigilant monitoring to detect disease progression. Incomplete phenotypes, as seen in this patient, challenge established diagnostic frameworks that often rely on hallmark features like ILD or arthritis. This highlights the importance of integrating histopathological, serological, and clinical findings to avoid diagnostic delays or misclassification. Additionally, this case supports revising diagnostic criteria to better account for atypical presentations, ensuring timely and accurate diagnosis.

Therapeutic challenges and management

The concurrent diagnosis of APS introduced significant therapeutic complexities. Long-term anticoagulation therapy with apixaban precluded the use of nonsteroidal anti-inflammatory drugs (NSAIDs), necessitating alternative strategies for managing musculoskeletal discomfort. Immunosuppressive therapy with prednisone and mycophenolate mofetil proved effective in controlling inflammation and improving muscle strength while minimizing the long-term side effects of corticosteroids. Mycophenolate mofetil was selected over azathioprine or methotrexate due to its efficacy in managing inflammatory myopathies and its favorable safety profile in the context of APS. The careful selection of immunosuppressive agents and the avoidance of medications that could exacerbate bleeding risk underscore the importance of individualized treatment plans in patients with complex comorbidities.

Physical therapy was integral to the patient’s management, focusing on resistance training and range-of-motion (ROM) exercises to improve muscle strength and prevent deconditioning, tailored to the patient’s functional limitations and overall condition. Hematology ensured appropriate anticoagulation management for the patient’s APS, balancing the risks of thrombosis and bleeding during immunosuppressive therapy. Dermatology played a key role in diagnosing polymyositis-like inflammatory myopathy by evaluating for autoimmune skin signs and interpreting muscle biopsy findings, guiding immunosuppressive treatment, and ongoing care. The multidisciplinary approach, involving rheumatology, hematology, and dermatology teams, facilitated coordinated care and close monitoring for treatment-related complications such as infection and organ toxicity. This case highlights the necessity of such collaborative efforts in managing complex autoimmune conditions.

Role of autoantibodies in disease pathogenesis

The overlapping presence of anti-PL-12, cytosolic 5' nucleotidase 1A, and anti-Ro52 antibodies raises important questions about the pathogenesis of polymyositis-like inflammatory myopathies. Anti-PL-12 antibodies, targeting alanyl-tRNA synthetase, are strongly associated with anti-synthetase syndrome, yet their role in isolated myositis without systemic features remains unclear [16]. Similarly, the presence of cytosolic 5' nucleotidase 1A antibodies, despite the absence of IBM-specific histological findings, suggests potential cross-reactivity or a subclinical overlap syndrome. Anti-Ro52 antibodies, linked to systemic autoimmune diseases, further complicate the immunological landscape and may reflect broader systemic immune activation. Current gaps in research include understanding the pathogenicity of these autoantibodies in atypical presentations. Future studies should focus on mechanisms of immune activation, cross-reactivity, and the development of predictive biomarkers for systemic involvement to inform diagnosis and management better.

These findings underscore the need for further research into the clinical implications of overlapping autoantibody profiles and conditions like APS and myositis, which may coexist as part of an overlap syndrome and share immune-mediated vascular injury mechanisms. The history of APS in this case highlights unique diagnostic and therapeutic challenges, particularly in balancing anticoagulation with immunosuppressive therapy for myositis. Both conditions involve vascular injury, with thrombotic vasculopathy in APS and complement deposition, such as C5b-9, in inflammatory myopathies. Understanding their role in disease pathogenesis, phenotypic variability, and therapeutic response could provide valuable insights into the management of complex autoimmune conditions and emphasize the importance of a multidisciplinary approach.

Clinical and research implications

This case underscores the importance of recognizing atypical presentations of anti-synthetase syndrome to avoid delays in diagnosis and treatment. It highlights the critical role of muscle biopsy in confirming autoimmune myopathy, particularly when serological and clinical findings are ambiguous. The findings also emphasize the need for ongoing research into the mechanisms driving phenotypic variability in autoimmune diseases.

Future studies should focus on the development of biomarkers for disease activity and organ-specific involvement, as well as the identification of therapeutic targets to optimize patient outcomes. The exploration of autoantibody profiles and their clinical significance could enhance diagnostic accuracy and refine treatment strategies for idiopathic inflammatory myopathies.

## Conclusions

This case highlights the diagnostic and therapeutic challenges of managing anti-synthetase syndrome presenting as an isolated polymyositis-like inflammatory myopathy. The absence of hallmark systemic features, such as ILD or mechanic’s hands, made diagnosis more complex. Muscle biopsy was critical in confirming immune-mediated muscle injury and excluding mimickers like inclusion body myositis. The concurrent diagnosis of APS further complicated management, requiring personalized immunosuppressive therapy which led to significant clinical improvement.

Multidisciplinary collaboration was essential in addressing this complex case and optimizing outcomes. This report expands the understanding of phenotypic variability in anti-synthetase syndrome, particularly in anti-PL-12-positive patients, and emphasizes the importance of early diagnosis and tailored treatment strategies. It also raises important questions about the prognostic implications of overlapping immune markers. Further research into targeted therapies and novel biomarkers is needed to guide treatment decisions and improve outcomes for patients with complex autoimmune myopathies.
